# Effects of comprehensive exercise training on frailty, negative emotions and physical functions of elderly patients with diabetes

**DOI:** 10.12669/pjms.40.6.7955

**Published:** 2024-07

**Authors:** Xiaoxiao Zhao, Xu Sha, Lanfeng Qi

**Affiliations:** 1 Xiaoxiao Zhao Department of Geriatrics, The No.2 Hospital of Baoding, Baoding 071000, Hebei, China; 2 Xu Sha Department of Geriatrics, The No.2 Hospital of Baoding, Baoding 071000, Hebei, China; 3 Lanfeng Qi Department of Endocrinology, The No.2 Hospital of Baoding, Baoding 071000, Hebei, China

**Keywords:** Comprehensive exercise training, Senile diabetes, Frailty, Negative Emotion, Physical Function, Treatment

## Abstract

**Objective:**

To evaluate the effects of comprehensive exercise training on frailty, negative emotions and physical functions of elderly patients with diabetes.

**Methods:**

This is a retrospective study. A total of 140 elderly patients with T2DM in The No.2 Hospital of Baoding were selected from December, 2021 to June, 2023 and randomly divided into two groups, with 70 patients in each group. The control group was given routine nursing and routine exercise education, and the study group was additionally given comprehensive exercise training. Tilburg frailty indicator (TFI), emotional status, physical functions, grip strength, fasting blood glucose and patient satisfaction were compared and analyzed between the two groups.

**Results:**

Before the intervention, TFI showed no significant differences between the two groups (*p>*0.05). After the intervention, physical, psychological and social frailty in the study group were significantly lower than those in the control group, with statistically significant differences (*p=* 0.00). SAS and SDS scores reduced significantly in the study group compared with those in the control group after the intervention, with statistically significant differences (*p=*0.00). After the intervention, the grip strength was significantly larger while the fasting blood glucose was significantly lower in the study group compared with those in the control group, with statistically significant differences (*p=*0.00). Patient satisfaction in the study group was higher than in the control group, with a statistically significant difference(*p*=0.03).

**Conclusion:**

Comprehensive exercise training for elderly patients with diabetes is beneficial to improving their frail state, negative emotions, blood glucose levels and physical functions. It has significant clinical application value.

## INTRODUCTION

With the continuous improvement of people’s living standards and dietary habits, the incidence of diabetes is increasing year by year, and Type-2 diabetes mellitus (T2DM) accounts for more than 90%, which is expected to increase significantly in the coming decades.^1^ A study^2^ has shown that negative emotions play a mediating role in senile diabetes and frailty, which can lead to cognitive impairment, reduce therapeutic compliance, and increase the risk of disability and death in the patients.^3^ Frailty is defined as reduced anti-stress ability while increased body vulnerability and disease susceptibility are caused by a decline in patients’ physiological reserve capability. At present, it has become a common complication of elderly diabetic patients. It has been demonstrated that^4^ the incidence of frailty in elderly diabetic patients is more than twice that of non-diabetic patients, and the readmission rate, case-fatality rate and disability rate are higher in elderly patients with diabetes combined with frailty.

Therefore, Nishikawa et al. believe that^5^ it is more important to focus on the improvement in the functional status of patients than simply controlling blood glucose. Frailty can lead to a decline in physical endurance, muscular strength, gait ability, balance ability and physical flexibility among the elderly.^6^ Providing comprehensive exercise training for the frail elderly plays an irreplaceable role in maintaining and improving the physical functions of patients. Comprehensive exercise training includes aerobic exercise, resistance exercise, balance training, stretching training (i.e., flexibility training) and coordination training.^7^ In recent years, it has been widely applied in the research on the prevention of declined physical functions and frailty. In the present study, comprehensive exercise training was carried out on elderly patients with diabetes, to evaluate the effects of comprehensive exercise training on frailty, negative emotions and physical functions of elderly patients with diabetes.

## METHODS

This is a retrospective study. A total of 140 elderly patients with T2DM in The No.2 Hospital of Baoding were selected from December, 2021 to June, 2023 and randomly divided into two groups, with 70 patients in each group. The general data showed no significant differences between the two groups, suggesting comparability (Table-I).

**Table-I:** Comparative analysis of general data between the study group and the control group (*χ̅*±*S*) n = 70.

Indicator	Study group	Control group	t/χ^2^	p
Age (year)	68.20 ± 7.86	69.48 ± 5.64	1.02	0.31
Male (n %)	42 (60%)	45 (64%)	0.38	0.54
Course of disease (year)	10.48 ± 3.72	10.63 ± 3.18	0.24	0.81
BMI (kg/m^2^)	26.57 ± 3.62	26.73 ± 3.39	0.25	0.80
TFI	11.53 ± 2.37	11.46 ± 2.85	0.15	0.88
***Underlying disease***				
Hyperlipidemia (n %)	12 (17%)	15 (21%)	0.43	0.51
Hypertension (n %)	23 (33%)	26 (37%)	0.31	0.58
CHD (n %)	9 (13%)	6 (9%)	0.69	0.41

*p*> 0.05.

### Ethical Approval:

The study was approved by the Institutional Ethics Committee of The No.2 Hospital of Baoding (No.:2023-015; date: June 15,2023), and written informed consent was obtained from all participants.

### Inclusion criteria:

- Meeting the diagnostic criteria for T2DM.^8^
- Tilburg frailty indicator (TFI) > 4.^9^
- Age >60 years.
- Willing to participate in the study and signing the informed consent.
- Complete clinical data.
- Able to cooperate in the research work.

### Exclusion criteria:

- Patients with severe visual and hearing impairment and communication difficulties.
- Patients with mental diseases, cognitive impairment and communication barriers.
- Patients suffering from other severe diseases who could not cooperate in the intervention.
- Patients at the end stage of diseases, with life expectancy < 6months.
- Patients with stress states such as trauma, surgery, acute and chronic infections recently.
- Patients with other conditions affecting the research results.
- Patients unsuitable for metformin and those requiring insulin to lower blood glucose have been excluded.

### Methods:

All patients were treated from the time of admission and the treatment period was 5-12 days, with evaluation starting 5 days after treatment. The control group was given routine nursing and routine exercise education. After admission, regular health education was provided to help patients and their families become familiar with the hospital condition as soon as possible. In addition, the patients received relevant treatment following medical advice, including oral or injectable hypoglycemics according to patients’ conditions. Every month, a telephone follow-up was conducted by the nurse in charge to inquire and record the patients’ exercise and other health conditions. If the patient has lactic acidosis, hypoglycemia and other adverse reactions in the course of medication, the doctor will timely adjust the hypoglycemic program and give symptomatic treatment.

The study group was additionally given comprehensive exercise training on the basis of the treatment in the control group. The main contents are as follows:

- A comprehensive exercise training team was established, responsible for program development; Nurses played a supervisory role, and regularly held health education lectures and conducted family follow-ups.
- Development of a personalized exercise program: At the initial intervention, the comprehensive training team evaluated the patients using the physical function scoring scale to determine their acceptable amount and intensity of exercise. Then, a personalized exercise training program was developed based on their age, underlying diseases and other actual conditions. Exercise atlas and self-monitoring diaries were distributed, and one-on-one guidance was provided to the patients during apparatus exercise training. For patients who could only adapt to low-intensity exercise, low-intensity exercises such as walking and jogging were carried out correctly.
- Training methods: The training included warm-up, balance exercise, stretching exercise, aerobic exercise and resistance exercise. Warm-up training was divided into five steps: shoulder, neck, head, waist, and marking time. A rest was taken for four minutes between each group of actions, and the intensity and strength of exercises were gradually increased as the intervention time..
- Health education: Sharing and exchanging meetings were organized regularly to share each other’s methods and experiences of exercises, learn from each other, and correct their own shortcomings. Elastic band resistance exercise and balance training were conducted with the assistance of the comprehensive exercise training team, and the intervention lasted for six months.

### Observation indicators:

***Comparative analysis of TFI:*** TFI was compared and analyzed between the two groups before and after the intervention. TFI is composed of a total of 15 items in three dimensions, including physical frailty (8 items: physical health, body weight, walking, balance, hearing, vision, grip strength, and fatigue), psychological frailty (4 items: memory, anxiety, depression, and coping ability), and social frailty (3 items: living alone, social relations, and social support). The score range of this scale was 0-15. When the score is > 4, the subjects are in a frail state. The higher the score, the severer the frailty.

- ***Comparative analysis of emotional status:*** The emotional changes of both groups were evaluated using the Self-Rating Anxiety Scale (SAS) and the Self-Rating Depression Scale (SDS) before and after the intervention, respectively.^10^ Lower scores indicated better emotional status.
- ***Comparative analysis of physical functions:*** Physical functions were assessed using the Short Physical Performance Battery (SPPB), including a balance test, a timed 4-m walking test and a timed chair-stand test, with a total score of 12. A score of 0-6, poor physical functions; a score of 7-9, moderate physical functions; a score of 10-12, good physical functions
- ***Comparative analysis of grip strength and fasting blood glucose:*** Grip strength was measured with the dominant hand three times at an interval of more than 15s, and the maximum was taken.

### Comparative analysis of patient satisfaction:

Patient satisfaction was compared and analyzed before and after intervention using the Patient Satisfaction Questionnaire Short Form (PSQ-18),^11^ including very satisfied, relatively satisfied, satisfied, uncertain, and dissatisfied. Total satisfaction = (very satisfied + relatively satisfied + satisfied)/total number of cases x 100%.

### Statistical analysis:

All data were statistically analyzed using SPSS 20.0. The measurement data were expressed as (*χ̅*±*S*). The sample size was estimated by 95% confidence interval. Inter-group analysis was performed using the two independent samples t-test, intra-group analysis using the paired t-test, and rate comparison using the χ^2^ test. *P*< 0.05 was considered statistically significant.

## RESULTS

Before the intervention, physical, psychological and social frailty showed no significant differences between the two groups(*p>* 0.05). After the intervention, the above indicators in the study group were significantly lower than those in the control group, with statistically significant differences (*p=* 0.00), as seen in Table-II.

**Table-II:** Comparative analysis of TFI between the two groups (*χ̅*±*S*) n = 70.

Indicator		Study group	Control group	t	p
Physical frailty	Before intervention	3.75 ± 0.24	3.68 ± 0.19	1.77	0.08
	After intervention*	3.12 ± 0.16	3.34 ± 0.14	6.87	0.00
Psychological frailty	Before intervention	4.06 ± 0.25	4.03 ± 0.31	0.58	0.56
	After intervention*	2.78 ± 0.22	3.08 ± 0.32	5.74	0.00
Social frailty	Before intervention	3.86 ± 0.18	3.89 ± 0.21	0.84	0.40
	After intervention*	2.80 ± 0.32	3.41 ± 0.43	8.82	0.00

* P < 0.05.

No significant differences were found in SAS or SDS scores between the two groups before intervention (*p>* 0.05). After the intervention, SAS and SDS scores reduced significantly in the study group compared with those in the control group, with statistically significant differences (*p=* 0.00) (Table-III).

**Table-III:** Comparative analysis of emotional status between the two groups before and after intervention (*χ̅*±*S*) n = 70.

Indicator		Study group	Control group	t	p
SAS	Before intervention	60.24 ± 8.06	60.73 ± 8.13	0.33	0.74
	After intervention*	53.08 ± 7.59	57.49 ± 6.15	3.50	0.00
SDS	Before intervention	61.40 ± 8.41	62.03 ± 7.92	0.42	0.67
	After intervention*	53.64 ± 6.81	57.79 ± 6.43	3.42	0.00

* P < 0.05.

Before the intervention, there were no significant differences in the balance test, the walking test or the timed chair-stand test between the two groups (*p>* 0.05). After the intervention, the above indicators in the study group were significantly superior to those in the control group, with statistically significant differences (*p=* 0.00), as presented in Table-IV.

**Table-IV:** Comparative analysis of physical functions between the two groups (*χ̅*±*S*) n = 70.

Indicator		Study group	Control group	t	p
Balance test	Before intervention	2.43 ± 0.12	2.40 ± 0.10	1.48	0.14
	After intervention*	3.68 ± 0.72	3.27 ± 0.58	3.44	0.00
Walking test	Before intervention	2.37 ± 0.61	2.40 ± 0.59	0.27	0.78
	After intervention*	3.52 ± 0.46	3.05 ± 0.33	6.43	0.00
Chair-stand test	Before intervention	2.75 ± 0.23	2.77 ± 0.21	0.50	0.62
	After intervention*	3.79 ± 0.72	3.46 ± 0.48	2.96	0.00

* P < 0.05.

Grip strength and fasting blood glucose presented no statistically significant differences between the two groups before intervention (*p>* 0.05). After the intervention, the grip strength was significantly larger while the fasting blood glucose was significantly lower in the study group compared with those in the control group, with statistically significant differences(*p=*0.00) (Table-V).

**Table-V:** Comparative analysis of grip strength and fasting blood glucose between the two groups before and after intervention (*χ̅*±*S*) n = 70.

Indicator		Study group	Control group	t	p
Grip strength (kg)	Before intervention	19.87 ± 5.86	20.06 ± 5.23	0.19	0.85
	After intervention*	24.47 ± 6.05	21.08 ± 5.71	3.16	0.00
Fasting blood glucose (mmol/L)	Before intervention	9.25 ± 2.47	9.18 ± 2.24	0.16	0.87
	After intervention*	6.60 ± 1.28	7.49 ± 1.52	3.47	0.00

* p < 0.05.

Patient satisfaction was 97% in the study group, and 87% in the control group. Patient satisfaction was higher in the study group than that in the control group, with a statistically significant difference (*p=* 0.03), as shown in Table-VI.

**Table-VI:** Comparative analysis of patient satisfaction between the two groups (*χ̅*±*S*) n = 70.

Group	Very satisfied	Relatively satisfied	Satisfied	Uncertain	Dissatisfied	Total satisfaction*
Study group	42	15	11	2	0	68 (97%)
Control group	38	10	13	6	3	61 (87%)
*χ* ^2^						4.83
*p*						0.03

## DISCUSSION

Our study showed that after intervention with comprehensive exercise training, physical, psychological and social frailty in the study group were significantly lower than those in the control group, with statistically significant differences (*p=*0.00). In addition, SAS and SDS scores reduced significantly in the study group compared with those in the control group after the intervention, with statistically significant differences (*p=*0.00), confirming that comprehensive exercise training can improve the frail state and negative emotions of elderly patients with diabetes. According to cause analysis, blood glucose level, insulin resistance, neuropathy, inflammatory reaction and oxidative stress may all be the influencing factors of senile diabetes-related frailty, of which sarcopenia is the core pathological basis of frailty.^12^

Currently, the routine exercise rehabilitation training used in clinical practice is mainly oral education, with lacking patient compliance, poor interventional effect, insignificant improvement in frailty, and the slow recovery of physical functions.^13^ Comprehensive exercise training is a personalized intervention program developed by a professional nursing team to help such patients undergo comprehensive and systematic exercise training, so as to make up for the shortcomings of conventional intervention.

Comprehensive exercise training integrates warm-up training, aerobic exercise, resistance exercise, balance training, and stretching training, which can improve mitochondrial function, increase slow-twitch fibers, and promote protein synthesis in skeletal muscle cells.^14^ Resistance exercise can mainly enhance myofibrillary protein synthesis, thereby increasing the size and cross-sectional area of myofibrils, especially fast-twitch fibers, and maintaining the quality and function of skeletal muscles.^15^ Balance training mainly improves the body’s balance ability and prevents adverse outcomes such as falls caused by frailty. Their combination effectively improves the patients’ frailty. Moreover, by strengthening the promotion of the importance of exercise, conducting relevant health education, developing personalized exercise programs, and encouraging exercise, the patients’ knowledge of and willingness to exercise increase, and their enthusiasm for exercise enhances, thus improving their negative emotions and enthusiasm.

Aerobic training in comprehensive exercise training mainly improves insulin resistance by increasing insulin receptors and insulin sensitivity. Resistance training can increase muscle strength and volume in a short period of time, and promote the transfer of intracellular glucose transporter 4 to the surface of skeletal muscle cell membranes, thereby increasing the uptake and utilization of glucose by skeletal muscles, and effectively reducing blood glucose levels.^16^ Balance training improves the flexibility and balance ability of the body, comprehensively improving patients’ physical functions. Grip strength, gait speed and SPPB are important indicators that reflect the physical functions of patients.^17^

In our study, it was confirmed that after the intervention, the balance test, the walking test and the timed chair-stand test in the study group were significantly superior to those in the control group, with statistically significant differences (*p*=0.00). After the intervention, the grip strength was significantly larger while the fasting blood glucose was significantly lower in the study group compared with those in the control group, with statistically significant differences (*p*=0.00). Peripheral insulin resistance originating from the skeletal muscles is the main driver for the development and progression of diabetes.^18^ Exercise improves glucose uptake of skeletal muscles through insulin-dependent and insulin-independent mechanisms, and regular exercise can continuously improve insulin sensitivity and glucose processing.^19^ Aerobic and resistance training programs can promote healthier skeletal muscles, adipose tissues, and hepatic and pancreatic functions, thereby enhancing muscle flexibility and the ability of glucose uptake.

Additionally, our study suggested that patient satisfaction in the study group was 97%, which was higher than 87% in the control group, with a statistically significant difference (*p=* 0.03). Chandratre et al.^20^ believe that comprehensive nursing intervention can improve the nurse-patient relationship, enhance patients’ psychological need satisfaction and sense of security, and increase their confidence in overcoming the disease so that the patients can actively cooperate with medical personnel for treatment, which is conducive to the recovery of the disease.

### Limitations:

It includes the small sample size, short follow-up time and few research indicators involved. In future clinical work, we will enlarge the sample size, increase the follow-up content, and add other research indicators such as the combination of diet and comprehensive exercise training, to further explore the optimal treatment for elderly patients with diabetes.

## CONCLUSION

Frail elderly patients with diabetes are characterized by serious negative emotions and frailty, low exercise compliance and poor physical functions. Comprehensive exercise training can significantly improve the exercise and physical functions of the patients, and reduce the influence of frailty and negative emotions, and it is an effective auxiliary treatment for elderly patients with diabetes.

### Authors’ Contributions:

**XZ:** Carried out the studies, participated in collecting data, drafted the manuscript, are responsible and accountable for the accuracy and integrity of the work.

**XS:** Performed the statistical analysis and participated in its design.

**LQ:** Participated in acquisition, analysis, or interpretation of data and drafting the manuscript.

All authors read and approved the final manuscript.
